# Expression of human eukaryotic initiation factor 3f oscillates with cell cycle in A549 cells and is essential for cell viability

**DOI:** 10.1186/1747-1028-5-10

**Published:** 2010-05-13

**Authors:** Ana E Higareda-Mendoza, Marco A Pardo-Galván

**Affiliations:** 1 División de Estudios de Posgrado, Facultad de Ciencias Médicas y Biológicas Dr. Ignacio Chávez, Universidad Michoacana de San Nicolás de Hidalgo, Morelia, Michoacán, México; 2 Departmento de Biología Molecular, Instituto de Investigaciones Químico-Biológicas, Universidad Michoacana de San Nicolás de Hidalgo, Morelia, Michoacán, México

## Abstract

**Background:**

Transcriptional and postranslational regulation of the cell cycle has been widely studied. However, there is scarce knowledge concerning translational control of this process. Several mammalian eukaryotic initiation factors (eIFs) seem to be implicated in controlling cell proliferation. In this work, we investigated if the human eIF3f expression and function is cell cycle related.

**Results:**

The human eIF3f expression has been found to be upregulated in growth-stimulated A549 cells and downregulated in G0. Western blot analysis and eIF3f promotor-luciferase fusions revealed that eIF3f expression peaks twice in the cell cycle: in the S and the M phases. Deregulation of eIF3f expression negatively affects cell viability and induces apoptosis.

**Conclusions:**

The expression pattern of human eIF3f during the cell cycle confirms that this gene is cell division related. The fact that eIF3f expression peaks in two cell cycle phases raises the possibility that this gene may exert a differential function in the S and M phases. Our results strongly suggest that eIF3f is essential for cell proliferation.

## Background

The initiation of translation in eukaryotes is a complex and multi-step process involving many translation initiation factors (eIFs). It is the rate-limiting and a major regulation step of mRNA translation. Translation initiation factor 3 (eIF3) is the largest of these factors and is involved in a number of different aspects of the initiation phase. eIF3 forms a stable complex with the 40S ribosomal subunit, which prevents premature association with the 60S subunit, binds the ternary complex eIF2-GTP-Met-tRNA to the 40S subunit [1] and promotes mRNA binding through interactions with the eIF4G subunit of the cap binding complex. Mammalian eIF3 has been purified [2] and consists of at least 13 non-identical subunits with molecular masses ranging from 35 to 170 kDa [3,4], but their individual functions have not been determined. The eIF3 subunit composition varies according to the organism, the simplest eIF3 subunit conformation (six subunits) [5], being identified in the budding yeast *Saccharomyces cerevisiae*, and the highest in humans (thirteen subunits from eIF3a to eIF3m) [6]. It has been shown that purified eIF3 complex from *S. cerevisiae *containing five subunits (eIF3a, eIF3b, eIf3c, eIF3g and eIF3i) can replace mammalian eIF3 in an in vitro assay for initiation [7]. This indicates a strong conservation of function of these subunits in eukaryotes, which form an active "core", with additional subunits that vary according to the organism, which may or may not be present in other eukaryotic eIF3s. For instance, eIF3j is present in mammals and yeast, but is lacking in plants [5]; eIF3d, eIF3e, eIF3f and eIF3h are present in mammals, plants and in the fission yeast *Schizosaccharomyces pombe*, but is absent in *S. cerevisiae*. It is proposed that distinct subclasses of eIF3 complexes, containing different combinations of core and non-core subunits, may regulate the translation of specific subsets of mRNAs [8]; as well as other suggested eIF3 activities [9].

eIF3f is a member of the Mov34 family. Members of this family contain an Mpr1/Pad N-terminal (MPN) motif and are involved in different cellular processes such as translation, transcription and proteasome regulation [10]. Although the role of eIF3f in the eIF3 complex has not been defined, shut-off experiments in *Schizosaccharomyces pombe *showed that in a long-term period, eIF3f is essential for viability, and that depleting this gene markedly decreases global protein synthesis [8]. It has also been suggested that eIF3f might be involved in muscle cell size [11]. *In vitro *studies using rabbit reticulocyte translation system indicate that in mammalian cells eIF3f is a negative regulator of translation [12,13]. Ectopic expression of this gene inhibits translation and overall cell protein synthesis in human A375 cells [13]. Shi *et al*. [13] also report that eIF3f is downregulated in several human tumours, and that its overexpression inhibited cell proliferation in melanoma and pancreatic cancer cells, inducing them to enter apoptosis.

Together, these previous findings suggest that eIF3f may be involved in the regulation of cell growth and proliferation. In the present study, we investigated the role of human eIF3f in cell cycle control. We report that eIF3f expression is related to A549 dividing cells and, that the expression profile of eIF3f during cell cycle is biphasic with one maximum expression peak in the early S phase and a second during mitosis. We show that eIF3f is essential for cell proliferation and its absence induces the cell to enter apoptosis.

## Results

### Human eIF3f expression is cell growth related

**Figure 1:**
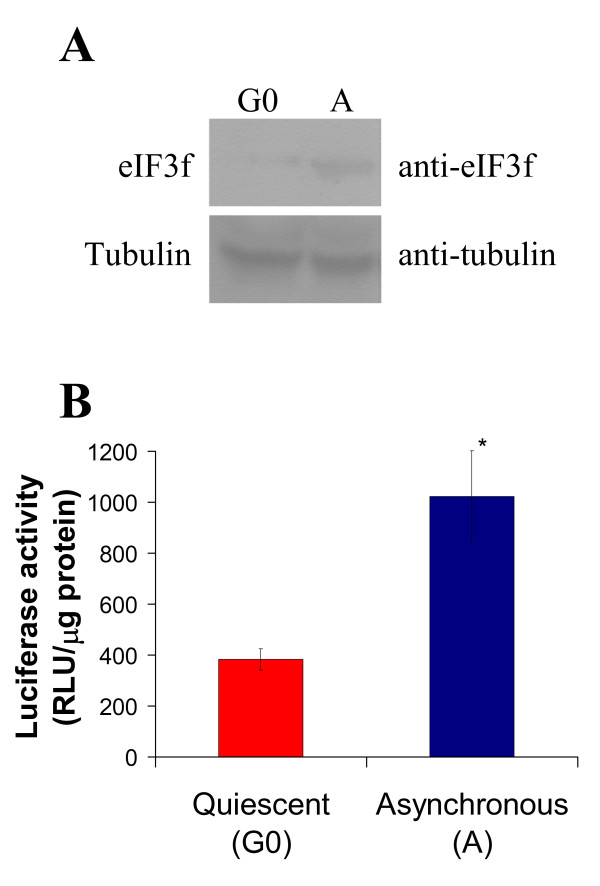
**Human eIF3f expression is cell growth related in A549 cells**. A) Western blot (WB) analysis of eIF3f expression in quiescent A549 cells (G0) and proliferating asynchronous cultures (A). Tubulin WB is shown to document equal protein loading. B) Promoter activity of the upstream sequences of eIF3f by promoter driven luciferase activity in quiescent A549 cells (G0) and proliferating asynchronous cultures (A). Luciferase activity with the empty vector pGL3 was below 10 RLU/μg of protein. Data expressed as mean ± SE; * p < 0.01 with respect to G0, n ≥ 3.

Modulation of serum concentration in cell culture media is a method that has been widely used to study regulatory mechanisms that control cell proliferation, which depends on microenvironmental signals such as the concentration of growth factors [14]. To induce cells to enter a non-dividing state (G0) by serum deprivation, A549 cells were grown according to Tzen *et al*. [14]. eIF3f protein abundance was determined by a Western blot experiment using a rabbit anti-eIF3f polyclonal antibody. Results are presented in Figure 1A (see additional file 1: Original Western blots for the data used in Figure 1A). As shown, eIF3f protein increased over two-fold in serum stimulated cells when compared to G0 cells. To confirm this data, G0 and serum stimulated cells were transiently transfected with a plasmid containing a luciferase reporter gene driven by the first kilobase of the human active eIF3f promoter (see section below). Results of three independent assays in serum-stimulated cells showed that luciferase activity increased in the same extent as that of the eIF3f protein when compared to G0 cells (Figure 1B). These results confirm that, in A549 cells, eIF3f expression is related to cell growth.

### A second eIF3f copy is present in human chromosome 2 and it is not transcriptionally active in A549 and 293-H human cells

**Figure 2:**
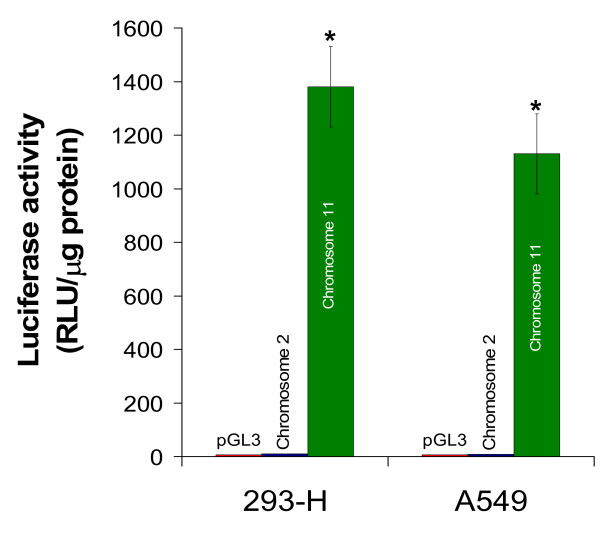
**Transcriptional activity of human eIF3f gene**. Promoter activity of the upstream sequence of eIF3f copy in chromosomes 2 is transcriptionally inactive in both 293-H and A549 asynchronous cultures, as exhibited by luciferase activity; in contrast, the eIF3f gene in chromosome 11 is active. Luciferase activity with the empty vector pGL3 was below 10 RLU/μg of protein. Data expressed as mean ± SE; * p < 0.01 with respect to control (pGL3), n ≥ 3.

Kojima and Okada [15] identified several potential 5'-inverted retrocopies, including eIF3f, and suggest that mRNA retrotransposition coupled with 5' inversion may be a mechanism to generate new genes distinct from parental genes. The human genome contains two complete coding sequences for eIF3f: one is located at chromosome 11 and contains 7 introns, and chromosome 2 contains a second intronless copy. There is a 97% identity between both coding sequences, which makes it difficult to differentiate between both putative mRNAs. Since the presence or abundance of eIF3f protein is an important parameter in the present study, we investigated which of these (or both) sequences are transcriptionally active, because of their probable contribution in overall eIF3f protein content. One kilobase upstream of the putative translation initiation site of each coding sequence was fused to the promotorless luciferase gene of pGL3 vector. A549 and 293-H cells were transfected with the above-mentioned genetic constructs and with the empty vector pGL3 as control, and luciferase activity was determined. The results of this experiment, shown in Figure 2, clearly demonstrate that the promoter of the chromosome 11 intron-containing eIF3f coding sequence is transcriptionally active in both cell lines, and that the promoter of the intronless coding sequence is not active in either one of them. We consider this approach as being the most direct method to determine promoter activity, since luciferase activity is highly sensitive and there is no endogenous luciferase activity.

### Human eIF3f expression oscillates during cell cycle

**Figure 3:**
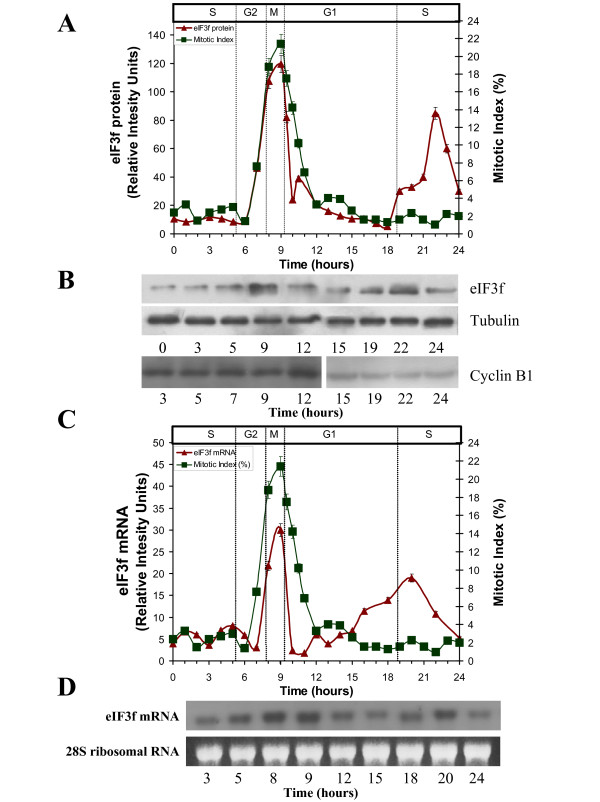
**Human eIF3f expression profile in A549 cells throughout the cell cycle**. A) Expression of eIF3f protein (relative intensity units normalized with tubulin, panel B of this Figure), and mitotic index (%, verified with Cyclin B1 expression shown in panel B of this Figure) in a 24 hour cell cycle in A549 cells after HU release (t = 0 h). B) WB analysis of eIF3f (top), tubulin (middle), and Cyclin B1 (bottom) along the 24 h cell cycle. All proteins visualized by their respective specific antibodies. Only some of the timepoints represented in panel A are shown in panel B. The same membrane was used for eIF3f and tubulin blots. A second membrane was used for Cyclin B1, where protein concentration load was verified by another tubulin blot (not shown). C) Expression of eIF3f mRNA (relative intensity units normalized with 28S ribosomal RNA), and mitotic index (%) in a 24 hour cell cycle in A549 cells after HU release (t = 0 h). D) Northern blot analysis of eIF3f mRNA (top) and 28S ribosomal RNA (bottom) along the 24 h cell cycle. Only some of the timepoints represented in panel C are shown in panel D.

**Figure 4:**
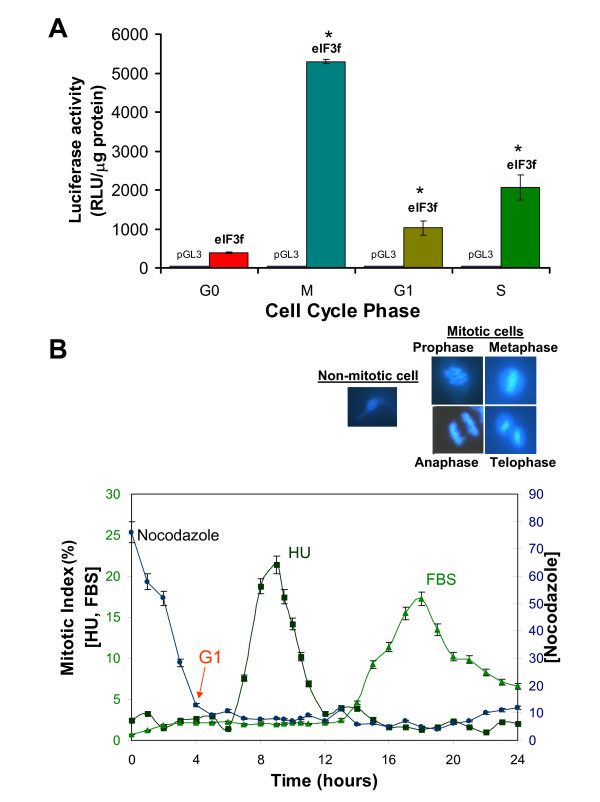
**Promoter activity of the upstream sequence of functional eIF3f in quiescent A549 cells (G0), M, G1, and S synchronous cultures**. A) Cell cultures in G0, M or S were transiently transfected with the promoter plasmid constructs and harvested 48 (for M and S cells) or 54 (for G0 cells) hours after transfection for luciferase activity assays. For cells in G1, cell cultures were transiently transfected with the promoter plasmid constructs for 20 hours, then arrested in M phase with nocodazole for 24 hours, and finally released from nocodazole to harvest cells 4 hours later for luciferase activity assays in G1. Luciferase activity with the empty vector pGL3 was below 10 RLU/μg of protein. Data expressed as mean ± SE; * p < 0.01 with respect to control (pGL3), n ≥ 3. B) Cell cycle distribution for nocodazole, HU and serum deprivation after release from arrest in M, S and G0, respectively. Cells were stained with DAPI at different timepoints to assess mitotic index. Micrographs of mitotic and interphase A549 cells are shown (top) at a magnification of 264×.

Once demonstrated that eIF3f expression is related to proliferation in A549 cells, we investigated its expression profile during the cell cycle. To evaluate eIF3f expression during the cell division process, eIF3f mRNA and protein abundance were determined at different cell cycle phases. A549 cells were synchronized with hydroxyurea (HU). eIF3f protein abundance for each sample was determined by Western blotting (Figure 3A, B). As shown in Figure 3 (see additional file 2: Original Western blots for the data used in Figure 3A, additional file 3: Original Western blots for the data used in Figure 3B, and additional file 4: Original Western blots for the Cyclin B1 data used in Figure 3B), eIF3f protein level peaks twice during the cell cycle: in the early S phase, and in Mitosis. Furthermore, eIF3f mRNA abundance during cell cycle progression was also determined for each sample by Northern blotting (Figure 3C, D) (see additional file 5: Original Northern blots for the data used in Figure 3C, and additional file 6: Original Northern blots for the data used in Figure 3D). These mRNA results show a similar expression pattern as in protein, peaking twice in the G2/M and G1/S transition cell cycle phases. To confirm these results, we evaluated eIF3f gene expression measuring its promotor activity in serum-starved G0 phase, with HU (S phase) and with nocodazole (M phase) treated cells (Figure 4A). Analysis of cell cycle distribution for each pharmacological agent used to arrest cells in the different cycle states is indicated in Figure 4B. Micrographs of mitotic and interphase cells are also represented to clearly distinguish between mitotic and non-mitotic cells using DAPI staining. Evidently, eIF3f promoter activity is upregulated during both cell cycle phases. This result strongly supports the possibility that the eIF3f function or functions are related to cell division.

### eIF3f overexpression affects cell viability in alveolar A549 cancer cells and induces apoptosis

**Figure 5:**
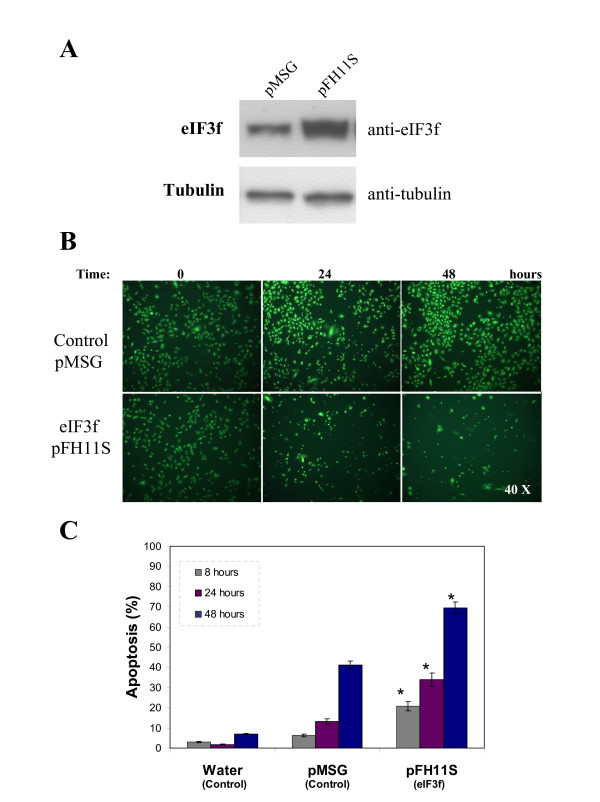
**Ectopic expression of human eIF3f in A549 cells**. A) WB analysis of eIF3f expression in transfected pMSG (control) and pFH11S (ectopic expression) A549 cultures. Tubulin WB is shown to document equal protein loading. B) Difference in cell growth in control and overexpressed eIF3f cultures after 24 and 48 hours of transfection. Timepoint cero represents cells before transfection. Cells were stained with acridine orange and ethidium bromide to visualize cell morphology and death by necrosis or apoptosis. Final magnification: 40×. C) Percent of apoptosis in control (water and pMSG) and overexpressed eIF3f (pFH11S) transfected cells, estimated from cell counts in microscopic fields similar to those shown in panel B. Data expressed as mean ± SE; * p < 0.01 with respect to control (water or pMSG), n ≥ 3.

We investigated the effect of eIF3f overexpression on alveolar adenocarcinoma cells, by transiently transfecting A549 cells with pFH11S containing a complete eIF3f cDNA, or with empty pMSG vector as control, and monitored its effect on cell viability. eIF3f overexpression was confirmed by Western blot analysis (Figure 5A) (see additional file 7: Original Western blots for the data used in Figure 5A). Cells overexpressing eIF3f show reduced viability (Figure 5B) (see additional file 8: Amplified micrographs for the data used in Figure 5B) and induction of apoptosis (Figure 5C).

### Endogenous eIF3f silencing by antisense RNA or siRNA inhibits cell growth and induces apoptosis in A549 cancer cells

**Figure 6:**
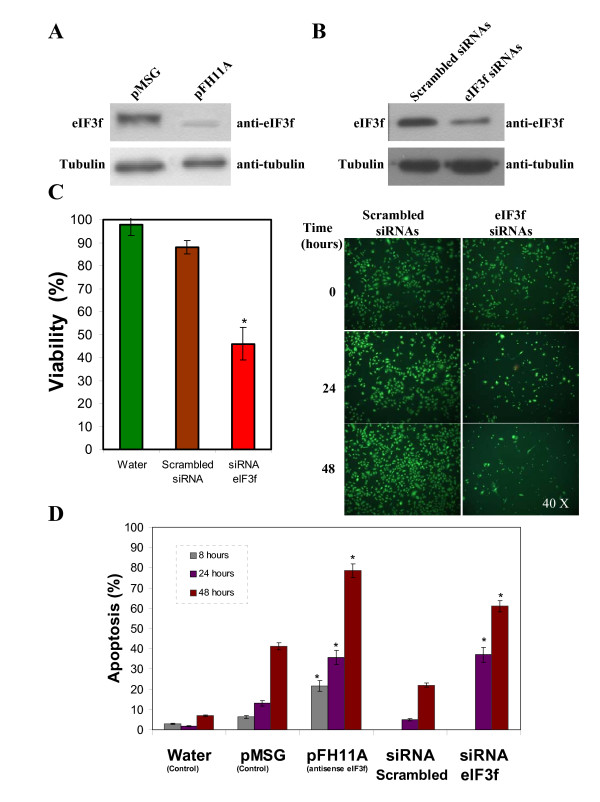
**Expression silencing of human eIF3f in A549 cells**. A) WB analysis of eIF3f expression in transfected pMSG (control) and pFH11A (antisense) A549 cultures. Tubulin WB is shown to document equal protein loading. B) WB analysis of eIF3f expression in transfected scrambbled (control) and eIF3f siRNAs A549 cultures. Tubulin WB is shown to document equal protein loading. C) Difference in cell growth in control (water and scrambled siRNAs) and eIF3f siRNA treated A549 cultures after 24 and 48 hours of transfection. Left: suspended and adherent cells stained with trypan blue to assess cell viability after 24 hours of transfection. Right: Cells were stained with acridine orange and ethidium bromide to visualize cell morphology and death by necrosis or apoptosis. Timepoint cero represents cells before transfection. Final magnification: 40×. D) Percent of apoptosis in control (water and pMSG), antisense eIF3f (pFH11A), scrambled siRNAs and eIF3f siRNAs transfected cells, estimated from cell counts in microscopic fields similar to those shown in panel C. Data expressed as mean ± SE; * p < 0.01 with respect to their respective control (water, scrambled siRNAs or pMSG), n ≥ 3.

To determine if eIF3f has a role on cell viability, A549 cells were transiently transfected with pFH11A (antisense), or with empty pMSG vector. eIF3f protein level was reduced in the antisense transfected cells with respect to the control as shown in (Figure 6A) (see additional file 9: Original Western blots for the data used in Figure 6A and 6B). The same effect was observed in siRNA treated cells (Figure 6B) (see additional file 10: Amplified micrographs for the data used in Figure 6C). Cell growth was monitored and, as shown, silencing eIF3f either by antisense or by siRNA significantly decreased cell viability (Figure 6C). Afterwards, using acridine orange and ethidium bromide staining and fluorescence microscopy, we investigated whether the decrease in cell viability by silencing eIF3f is associated with increased apoptosis. We observed significantly increased apoptosis in cells transfected with antisense-eIF3f or siRNA-eIF3f (Figure 6D). These results show that eIF3f silencing affects cell viability and induces apoptosis in A549 cells.

## Discussion

Proper execution of the cell cycle requires the expression and activation of key proteins at specific times. Transcriptional regulation and post-translational modifications, such as phosphorylation and proteolysis, are important for cell-cycle progression and have been studied extensively. However, much less is known about cell-cycle-specific translational control. Nevertheless, important data has been obtained involving initiation of translation, the rate limiting step of the process, in the control of cell proliferation. Expression of eIF4E is commonly elevated in many human cancers and, frequently, it induces cellular transformation, tumorigenesis and metastasis [16,17] selectively enhancing the translation of mRNAs of potent growth regulatory proteins such as Cyclin D [18].

Several investigations strongly suggest that eIF3 subunits are involved in cell cycle regulation [19]. For example, elevated expression of eIF3a might be associated with oncogenesis in some cancers, and it is possibly implicated in regulating the expression of ribonucleotide reductase [19] and p27 protein [20]. Downregulating the expression of eIF3e by RNAi inhibited mitosis in HeLa cells [21]. Also, it has been reported that eIF3k binds to Cyclin D3, a member of the Cyclin D family that mediates G1 phase progression [22].

Although eIF3f has been implicated in cell proliferation its role in this process has not been elucidated. In the present work we report that the deregulation of the expression of eIF3f markedly affects cell proliferation. eIF3f overexpressing in A549 cells show a significantly increase in apoptosis compared to those transfected with the vector alone (Figure 5). This result is in agreement with that found in melanoma and pancreatic cancer cells [13]. Downregulation of eIF3f expression by antisense-eIF3f markedly increased apoptosis in A549 cells (Figure 6A). To confirm that result, we decided to transfect A549 cells with siRNA-eIF3f as a different and independent experimental strategy. The results of this experiment were similar to those obtained by antisense-eIF3f (Figure 6B). These results clearly show that eIF3f is essential for the viability of A549 lung adenocarcinoma cells and that silencing of this gene promotes apoptosis; in agreement with this result, we found that downregulation of eIF3f expression in plant cells also induced apoptosis (unpublished data). Our data show that deregulation of eIF3f in A549 cells induce apoptosis by means of a caspase-9 independent pathway (data not shown). Similarly, Shi et al. [13] report that eIF3f overexpression in melanoma and pancreatic cancer cells induce apoptosis through a caspase-3 independent pathway. The fact that both knocking down and ectopically overexpressing eIF3f suppressed cell proliferation and induced apoptosis, suggests that deregulation of eIF3f expression is not tolerated by the cell. This may be explained by the specific expression seen during cell cycle progression. Since the cell cycle progression implies a coordinated and controlled series of events, the increase or decrease in eIF3f expression may affect the coordination of events specific to a cell cycle phase, where it is needed as a negative regulator of translation or required to be absent.

To determine if eIF3f is associated to cell division, we investigated weather there was a difference in eIF3f expression between dividing and G0/G1 arrested cells. A significantly decrease in eIF3f protein was found in G0 cells (Figure 1A), and this result was corroborated measuring eIF3f-promotor activity (Figure 1B). Clearly, eIF3f expression is associated with cell division. Recently, it was reported that eIF3a expression is cell growth-related, unlike the core subunit eIF3b and the non-core subunit eIF3d [23]. Interestingly, eIF3a expression increases during growth in NIH3T cells, in a similar extent as does eIF3f in A549 cells.

We investigated the relationship between eIF3f expression and cell cycle progression. We measured human eIF3f protein abundance and eIF3f promoter activity profile through different cell cycle phases in synchronized A549 cells, as shown in Figure 3. Surprisingly, eIF3f expression oscillates during the cell cycle following a biphasic profile, with one peak in the S phase and another one in the M phase. Recently, it was shown that eIF3a expression peaks in S phase in NIH3T cells [23]. Biphasic translation at G1/S and G2/M is observed in ornithine decarboxylase in HeLa cells [24]. In this case, ornithine decarboxylase mRNA peaks only at G1/S, so at G2/M the control of expression is exerted at the translational level. In fission yeast (*S. pombe*) and plant cells (tobacco BY2 cell line), eIF3f expression peaks once during the cell cycle in the G2 and M phases, respectively (unpublished data), which suggests that the M phase expression peak in human A549 cells represents the original eIF3f function common to eukaryotes, and that the S phase peak in human cells was later acquired. It remains to be elucidated if eIF3f has the same function in both cell cycle phases, as well as if other eIF3 subunits are growth and cell cycle related in A549 cells and in other cell types. Several studies show that eIF3f inhibits protein synthesis [8,13]. Although the inhibitory effect of eIF3f on translation is robust, it is not a total protein synthesis inhibition: it diminishes protein synthesis by 60-70%, remaining a 30-40%. Our interpretation is that eIF3f is not an inhibitor, but a translational modulator that selects mRNAs at specific cell cycle phase timepoints. This observation is supported by previous studies where *Arabidopsis thaliana *translation initiation factor subunit eIF3h apparently selects specific mRNAs through 5'mRNA leader sequences [25], and *S. pombe *non-core subunits eIF3m and eIF3e define distinct translation initiation factor 3 complexes, translating different sets of mRNAs [8]. Fan and Penman [26] report that protein synthesis is greatly inhibited in the G2/M and M phase in mammalian cells; in this work we report that eIF3f is expressed in the M phase of the cell cycle, so it is reasonable to suggest that eIF3f contributes to this protein synthesis inhibition observed in the M phase. We speculate that if eIF3f is a protein synthesis modulator, then mRNAs that should not be translated during M cell cycle phase are selected and transcribed during endogenous eIF3f knock down, contributing to apoptosis induction.

Finally, the human genome contains two complete eIF3f gene sequences, one with 7 introns and another one with no introns. The human intron-containing sequence has a similar genetic organization as that in most organisms (data not shown), while the intronless one has characteristics of a retrocopy [15,27]; both sequences having a 97% identity between them. Retroduplication is commonly thought to generate nonfunctional gene copies, because they are expected to lack regulatory elements that could promote retrocopy transcription. However, different studies have shown that transcription of retrocopies may not be rare [27-29]. Because there is a second eIF3f expression peak in A549 cells, it was important to know if one or both eIF3f copies were expressed. As Figure 2 shows, when two different human cell lines, A549 and 293-H, were transfected with either copy's promotor, only that of the intron-containing copy of eIF3f was transcriptionally active: thus, the intronless copy is most likely a retropseudogene.

## Conclusions

In this work we provide evidence that relates translation with control of the cell cycle through eIF3f expression and function. eIF3f function is related to the mitotic phase in human A549 cells, and is essential for cell viability. eIF3f abundance peaks in the S phase in human A549 cells and this increase seems not to be related to the presence of an intronless eIF3f copy, but to regulatory elements of the chromosome 11 eIF3f gene promoter.

## Methods

### Materials

Monoclonal antibodies against tubulin and caspase-9 were from Sigma Aldrich (ST. Louis, MO) and Upstate (Lake Placid, NY), respectively. Rabbit polyclonal antibody against eIF3f was obtained from Global Peptide Services, LLC (Fort Collins, CO) and later from Biolegend (San Diego, CA). Mouse anti-Cyclin B1monoclonal antibody and horseradish peroxidase-conjugated anti-rabbit and anti-mouse secondary antibodies were from Santa Cruz Biotechnology, Inc., Santa Cruz, CA). The pBluescript SK(-) vector is from Stratagene (La Jolla, CA). The pGEM-T Easy and pGL3 basic plasmids, restriction enzymes, DNA and RNA molecular weight markers, and RNA polymerases were from Promega (Madison, WI). The pMSG vector, GenomicPrep™ Cells and Tissue DNA Isolation Kit, Protease Inhibitor Mix, protein molecular weight markers, Hybond-P PVDF Membrane, Amersham Hybond™-N+, and the Enhanced Chemiluminescence (ECL) system for Western blot analysis were from GE Healthcare Life Sciences (Piscataway, NJ). 293-H cell line, 293 SFM II medium, synthesized primers, the pSL301 vector, LipofectAMINE 2000, TRIzol^®^, and Quant-iT™ RiboGreen RNA Assay Kit were from Invitrogen (Carlsbad, CA). DIG RNA Labeling Kit (SP6/T7), DIG Luminescent Detection Kit and Lumi-Film Chemiluminescent Detection Film were from Roche (Indianapolis, IN). All other reagents were of molecular biology grade and obtained from Sigma Aldrich (Saint Louis, MO).

### Cell culture

A549 cells, a human lung adenocarcinoma epithelial line, were obtained from ATCC (designation CCL-185) and used for all experiments. The cells were cultured in MEM supplemented with 10% heat-inactivated fetal bovine serum (FBS) at 37°C in a humidified atmosphere with 5% CO2. A549 cells are adherent and grow with a doubling time of approximately 22 hours. 293-H cells, clonal isolates derived from transformed embryonal human kidney cells, were cultured in suspension with 293 SFM II medium supplemented with 2 mM L-glutamine at 37°C in a humidified atmosphere with 8% CO2. A549 and 293-H cells were routinely passaged twice per week to maintain them in a logarithmic growth phase.

### Plasmid constructs

The pGL3 basic plasmid was used as the luciferase reporter expression vector, where different fragments of the human eIF3f promoter gene were introduced. Fragments were amplified by PCR from A549 genomic DNA, extracted with the GenomicPrep Kit. For the intron-containing gene [Locus NP_003745], mapped on chromosome 11p15.4, we used the 11:7947867-11:7948866 fragment to generate a plasmid, called pF11P1K, that encompasses 1 kb of genomic DNA corresponding to the promoter (Forward Primer: ACATAGTGATAAAAGCTTTGACTTAATAAC, Reverse Primer: GCCGGTGTGGCAAGCTTGTCGAGAAAGAAGG). For the intronless copy [NCBI removed record XM_290345], mapped on chromosome 2p16.1, we used the 2:58331072-2:58332078 fragment to generate a plasmid, called pF02P1K, that also encompasses 1 kb of genomic DNA corresponding to the promoter region of the eIF3f intronless copy (Forward Primer: TGTGGAGGATGTATTGAGCTAAAAC, Reverse Primer: TCTTGTCGAGATCTAGCTTTACTTTCAAAC). The pMSG plasmid with the leaky inducible (dexamethasone) MMTV LTR promoter was used to generate eIF3f gene expression vector, called pFH11S, and eIF3f antisense vector, called pFH11A. Total RNA was extracted from A549 cells with TRIzol, according to the manufacturer's instructions. eIF3f gene sequence [NCBI Entrez Gene 86652: Locus NP_003745] was amplified by RT-PCR using forward primer TTCTCGACAAGATGGCCACACCGGCG and reverse primer TCACAGGTTTACAAGTTTTTCATTGAG. Amplification product was subcloned in the pBluescript SK(-) vector (plasmid construct pSK11F) and orientated into the pMSG plasmid to generate the sense and antisense constructs. All constructs were verified by sequencing (Macrogen Corp, Rockville, MD). The verified antisense targeting sequence of eIF3f is 5'---ttctcgacaagATGgccacaccggcggtaccagtaagtgctcctccggccacgccaaccccagtcccggcggcggccccagcctca

gttccagcgccaacgccagcaccggctgcggctccggttcccgctgcggctccagcctcatcctcagaccctgcggcagcagcggctg

caactgcggctcctggccagaccccggcctcagcgcaagctccagcgcagaccccagcgcccgctctgcctggtcctgctcttccagg

gcccttccccggcggccgcgtggtcaggctgcacccagtcattttggcctccattgtggacagctacgagagacgcaacgagggtgct

gcccgagttatcgggaccctgttgggaactgtcgacaaacactcagtggaggtcaccaattgcttttcagtgccgcacaatgagtcag

aagatgaagtggctgttgacatggaatttgctaagaatatgtatgaactgcataaaaaagtttctccaaatgagctcatcctgggctg

gtacgctacgggccatgacatcacagagcactctgtgctgatccacgagtactacagccgagaggcccccaaccccatccacctcact

gtggacacaagtctccagaacggccgcatgagcatcaaagcctacgtcagcactttaatgggagtccctgggaggaccatgggagtga

tgttcacgcctctgacagtgaaatacgcgtactacgacactgaacgcatcggagttgacctgatcatgaagacctgctttagccccaa

cagagtgattggactctcaagtgacttgcagcaagtaggaggggcatcagctcgcatccaggatgccctgagtacagtgttgcaatat

gcagaggatgtactgtctggaaaggtgtcagctgacaatactgtgggccgcttcctgatgagcctggttaaccaagtaccgaaaatag

ttcccgatgactttgagaccatgctcaacagcaacatcaatgaccttttgatggtgacctacctggccaacctcacacagtcacagat

tgcactcaatgaaaaacttgtaaacctgTGA---3'.

### siRNA design and production

Design and production of siRNAs against eIF3f was carried out according to Kittler *et al*. [30]. Briefly, eIF3f sequence was analyzed in the DEQOR web-program [31] to select a 400 to 600 bp sequence for the design and quality control of siRNAs. The sequence from 529 to 1020 bp was amplified by PCR from the pFH11S vector using forward primer CATGACATCACAGAGCAC and reverse primer CAGGTAGGTCACCATCAA. The siRNA targeting sequence of eIF3f is 5'---catgacatcacagagcactctgtgctgatccacgagtactacagccgagaggcccccaaccccatccacctcactgtggacacaagt

ctccagaacggccgcatgagcatcaaagcctacgtcagcactttaatgggagtccctgggaggaccatgggagtgatgttcacgcctct

gacagtgaaatacgcgtactacgacactgaacgcatcggagttgacctgatcatgaagacctgctttagccccaacagagtgattggac

tctcaagtgacttgcagcaagtaggaggggcatcagctcgcatccaggatgccctgagtacagtgttgcaatatgcagaggatgtactg

tctggaaaggtgtcagctgacaatactgtgggccgcttcctgatgagcctggttaaccaagtaccgaaaatagttcccgatgactttga

gaccatgctcaacagcaacatcaatgaccttttgatggtgacctacctg---3'.

The amplified sequence was cloned into the pGEM plasmid to generate the pF11SIR vector. dsRNA were obtained by *in vitro *transcription of the eIF3f fragment sequence with T7 and T3 RNA polymerases. siRNAs against eIF3f were obtained by digestion of dsRNA with RNase III, generating a mixture of siRNAs from 25 to 30 pb. Scrambled siRNAs were obtained by *in vitro *transcription of the MCS of vector pSL301 with SP6 and T7 RNA polymerases and further digestion with RNase III. siRNAs were cleaned with Q-sepharose columns.

### Cell synchronization

Cells were accumulated in G0 by serum deprivation (0.1% FBS) for 48 hours [14]. Synchronization and accumulation of cells in M was achieved by adding 0.4 μg/mL of nocodazole to the culture media [32]. Synchronization in S phase was done by adding of 0.8 mM HU to the culture media [33]. After 24 hours of synchronization with HU or nocodazole, samples were processed to determine parameters in S or M phase, respectively. For sample taking at specific timepoints, cells were washed 2 times with phosphate-buffered saline (PBS, pH 7.4), another 2 times with MEM and refed with fresh medium. Cells were accumulated in G1 by releasing cells synchronized in M for 24 hours, washing twice with PBS and MEM, adding MEM and incubating for 4 hours. Cell synchrony was microscopically (LEICA DM IL FLUO, Wetzlar, Germany) assessed by determining the mitotic index on cells fixed with 1% formaldehyde/0.2% glutaraldehyde for 5 min at room temperature [34], and stained with 4',6-diamidino-2-phenylindole (DAPI). Each timepoint was assessed in triplicate, and at least 400 cells were scored in each sample. To confirm mitotic index, Cyclin B1 expression was determined by Western blot analysis at specific timepoints: from serum-starved cells (Q: quiescence), mitotic cells (M), proliferating asynchronous cells (A), and serum stimulated cells at 0, 3, 5, 7, 9, 12, 15, 19, 22 and 24 hours after HU release [35-37].

### Northern-blot analysis

Total RNA was extracted from A549 cells with TRIzol, according to the manufacturer's instructions. The resulting RNA pellet was air-dried, resuspended in nuclease-free water, and treated with amplification-grade DNase I. RNA was quantified using a spectrophotometer (Jenway, Burlington, NJ); A260/280 readings between 1.8 and 2.0 were used to ensure purity. Total RNA was also quantified with a VersaFluor fluorometer (Bio-Rad, Hercules, CA) using Quant-iT™ RiboGreen RNA Assay Kit, according to the manufacturer's instructions. Agarose/formaldehyde denaturing gel electrophoresis, as described by Sambrook and Russel [34], was performed with 10 μg of total RNA, and intact 18S and 28S ribosomal RNA were verified. To compare the RNA loaded in each lane, 28S ribosomal RNA was quantified and used to normalize eIF3f mRNA data. For quantification purposes, 28S ribosomal RNA densitometric measurements were performed using the Kodak Digital Science Electrophoresis Documentation and Analysis System 100 for Windows (Rochester, NY). An upward capillary transfer of RNA from the gel to a nylon membrane (Amersham Hybond™-N+) [34] was performed, and RNA was fixed on the membrane by baking 2 hours at 80°C. According to the manufacturer's specifications, a complementary antisense single-stranded RNA probe of eIF3f was produced with the DIG RNA Labeling Kit (T7) using the plasmid construct pSK11F as the template: eIF3f cDNA subcloned in the pBluescript SK(-) vector in a antisense orientation with respect to the T7 RNA polymerase site. Prehybridization, RNA:RNA hybridization and high stringency post-hybridization washes of blots were performed according to the DIG Application Manual for Filter Hybridization (Roche). Hybridization bands were visualized by DIG Luminescent Detection Kit using Lumi-Film Chemiluminescent Detection Film.

### Western-blot analysis

For Western blot analysis, cells were scraped from dishes and cellular protein extracts were prepared by homogenization in ice cold lysis buffer (25 mM Tris pH 7.8, 15 mM MgSO_4_, 4 mM EGTA, 1% Triton X-100 and 1 mM DTT) containing a protease inhibitor mix. 15 μg of protein were separated per lane by 10% SDS-PAGE gel electrophoresis. After electrophoretic transfer of proteins to a polyvinylidene difluoride (PVDF) membrane, eIF3f, Cyclin B1 or caspase-9 specific bands were detected by reaction with a rabbit antibody against eIF3f, a mouse antibody against caspase-9, followed by horseradish peroxidase-conjugated anti-rabbit or anti-mouse secondary antibody, respectively. Bands were visualized by enhanced chemiluminescence (ECL). Immunoblots were stripped using mild antibody stripping solution and re-probed with a mouse anti-tubulin antibody. For quantification purposes, densitometric measurements were performed using the Kodak Digital Science Electrophoresis Documentation and Analysis System 100 for Windows (Rochester, NY). All eIF3f values were normalized to tubulin levels.

### Transient transfections

Transient transfections were performed using the cationic lipid, LipofectAMINE 2000, according to the manufacturer's specifications. All of these experiments were performed in 6-well tissue culture plates with cells plated to reach 60-70% confluence on the day of transfection. Nucleic acid concentration for transfections with constructed and empty plasmids (vector control) was 2 μg, and 3 μg for the siRNAs. Transfection times varied according to each experiment. Cells were harvested 8, 24 and 48 hours after transient transfection with siRNAs, eIF3f sense and antisense constructs, and control plasmids. For transfections with pMSG and derived constructs, 0.1 mM dexamethasone was added to the culture medium. For luciferase activity assays with cells in G1, cell cultures were transiently transfected with the promoter plasmid constructs for 20 hours, then arrested in M phase with nocodazole for 24 hours, and finally released from nocodazole to harvest cells 4 hours later (G1 cells). For luciferase activity assays with cells in M or S, first cell cultures were transiently transfected with the promoter plasmid constructs for 24 hours, then these cultures were arrested with nocodazole or hydroxyurea for 24 hours, to finally harvest transfected cells in M or S phase, respectively. To determine luciferase activity in G0, cell cultures were transiently transfected with the promoter plasmid constructs for 6 hours, and then cultures were accumulated in G0 by serum deprivation for 48 hours after which transfected cells were harvested for luciferase activity assays.

### Luciferase activity assay

Promoter activity was determined by measuring the luciferase activities of cell lysates 48 hours after transfection with empty pGL3, pF11P1K or pF02P1K plasmids. Briefly, cells were lysed in ice cold lysis buffer (25 mM Tris pH 7.8, 15 mM MgSO_4_, 4 mM EGTA, 1% Triton X-100 and 1 mM DTT). Each clarified cell lysate was first assayed for total protein, using the Bio-Rad Protein Assay, according to the manufacture's instructions (Bio-Rad, Hercules, CA). After adjusting samples for equivalent protein concentration in lysis buffer, samples were assayed for luciferase activity as described by Sambrook and Russel [34] with a Sirius Single Tube V3.1 Luminometer (Berthhold, Huntsville, AL). Luciferase activity was normalized to the respective total cellular protein.

### Determination of cell viability

Adherent cells were released from the plate with Trypsin-EDTA Solution (1×) and collected. Floating cells were also recovered and included with the formerly adherent cells for cell viability with the trypan blue exclusion method. Percent of cell viability was determined microscopically (LEICA DM IL, Wetzlar, Germany) by counting triplicate samples of 200 to 400 cells in a hemotocytometer chamber.

### Determination of apoptosis and necrosis

Adherent cells were washed twice with PBS and stained with a mixture of 4 μg/ml acridine orange and 4 μg/ml ethidium bromide in PBS [38] for 5 minutes. Cells were fixed with 1% formaldehyde/0.2% glutaraldehyde for 5 min at room temperature [34] and visualized by epifluoresence microscopy (LEICA DM IL FLUO, Wetzlar, Germany) at a magnification of 40×. Viable (normal, green nuclei), early apoptotic (condensed, green nuclei), late apoptotic (condensed, red nuclei), and necrotic (normal, red nuclei) cells were counted. At least 400 cells were scored in triplicate for each sample.

### Statistical analysis

All experiments were repeated at least three times. Results of multiple experiments are expressed as mean ± standard error (SE). Analysis of Student's t test was used to assess the differences between means. A P < 0.05, with respect to control experiments, was accepted as statistically significant.

## Competing interests

The authors declare that they have no competing interests.

## Authors' contributions

AEHM participated in the design of the study, carried out the molecular genetic and cellular studies, performed the statistical analysis and drafted the manuscript. MAPG conceived the study and participated in its design and coordination and helped to draft the manuscript. All authors read and approved the final manuscript.

## Acknowledgements

We would like to thank Y.E. Guzmán-Infante, A. Mendoza-Pineda, D.A. Bolaños-Cornejo and A. Aguilera-Méndez for technical assistance. We would also like to thank S. Zárate for critically reading the manuscript. A.E. Higareda-Mendoza was a recipient of a CONACyT Doctoral Scholarship. This research was supported by CIC Research Programs 2.15 and 16.3 from the Universidad Michoacana de San Nicolás de Hidalgo, and the Mixed Fund CONACyT-Goverment of the State of Michoacan MICH-2009-C05-116168.

## Supplementary Material

Additional file 1 **Original Western blots for the data used in Figure **1A. Original Western blots (WB) for the analysis of eIF3f expression in quiescent A549 cells (G0) and proliferating asynchronous cultures (A). The first WB (left) shows eIF3f expression, detected with a polyclonal rabbit anti-human eIF3f antibody (primary antibody) and HRP-conjugated goat anti-rabbit antibody (secondary antibody). To document equal protein loading, the second WB (right) shows tubulin expression, detected with a monoclonal mouse anti-human beta-tubulin antibody (primary antibody) and HRP-conjugated goat anti-mouse antibody (secondary antibody). Click here for file

Additional file 2 **Original Western blots for the data used in Figure **3A. Original Western blots (WB) for the analysis of eIF3f and tubulin expression in A549 synchronous cultures. The left WBs show eIF3f expression, detected with a polyclonal rabbit anti-human eIF3f antibody (primary antibody) and HRP-conjugated goat anti-rabbit antibody (secondary antibody). To document equal protein loading, the WB at the right show tubulin expression, detected with a monoclonal mouse anti-human beta-tubulin antibody (primary antibody) and HRP-conjugated goat anti-mouse antibody (secondary antibody). Numbers represent the time in hours after HU release in A549 cultures. (mw: protein molecular weight). Click here for file

Additional file 3 **Original Western blots for the eIF3f and tubulin data used in Figure **3B. Original Western blots (WB) for the analysis of eIF3f and tubulin expression in A549 synchronous cultures. The top WB shows eIF3f expression, detected with a polyclonal rabbit anti-human eIF3f antibody (primary antibody) and HRP-conjugated goat anti-rabbit antibody (secondary antibody). To document equal protein loading, the WB at the bottom shows tubulin expression, detected with a monoclonal mouse anti-human beta-tubulin antibody (primary antibody) and HRP-conjugated goat anti-mouse antibody (secondary antibody). Numbers represent the time in hours after HU release in A549 cultures. Click here for file

Additional file 4 **Original Western blots for the Cyclin B1 data used in Figure **3B. Original Western blots (WB) for the analysis of Cyclin B1 expression in A549 synchronous cultures. The left WBs show Cyclin B1 expression, detected with a monoclonal mouse anti-Cyclin B1 antibody (primary antibody) and HRP-conjugated goat anti-mouse antibody (secondary antibody). To document equal protein loading, the WBs at the right show tubulin expression, detected with a monoclonal mouse anti-human beta-tubulin antibody (primary antibody) and HRP-conjugated goat anti-mouse antibody (secondary antibody). Numbers represent the time in hours after HU release; M represents expression in mitotic A549 cells, by accumulating cells with nocodazole; A represents expression in A549 proliferating asynchronous cultures; G0 represents expression in quiescent A549 cells, by accumulating cells with serum deprivation; and mw, protein molecular weight. Click here for file

Additional file 5 **Original Northern blots for the data used in Figure **3C. A) Original Northern blots (NB) for the analysis of eIF3f mRNA expression in A549 synchronous cultures. The NBs show eIF3f mRNA expression, detected with a complementary antisense single-stranded RNA probe of human eIF3f. To document equal RNA loading and to normalize the expression of eIF3f mRNA, the relative intensity units of the 28S ribosomal RNA bands were determined. B) Total RNA gel photographs, corresponding to the NBs shown in panel A, and taken with a Hoefer Photoman Polaroid Camera (Hoefer Inc., Holliston, MA). Numbers represent the time in hours after HU release, and A represents expression in A549 proliferating asynchronous cultures. Click here for file

Additional file 6 **Original Northern blots for the data used in Figure **3D. Original Northern blot (NB) for the analysis of eIF3f mRNA expression in A549 synchronous cultures. The NB (top) shows eIF3f mRNA expression, detected with a complementary antisense single-stranded RNA probe of human eIF3f. To document equal RNA loading and to normalize the expression of eIF3f mRNA, the relative intensity units of the 28S ribosomal RNA bands were determined. Total RNA gel photograph (bottom) corresponds to the NB shown above, and taken with a Hoefer Photoman Polaroid Camera (Hoefer Inc., Holliston, MA). Numbers represent the time in hours after HU release. Click here for file

Additional file 7 **Original Western blots for the data used in Figure **5A. Original Western blots (WB) for the analysis of eIF3f expression in A549 cultures transfected with pMSG (control) and pFH11S (ectopic expression). A549 cells were lysed 48 hours after transfection and proteins were transferred to a PVDF membrane after separation by SDS-PAGE gel electrophoresis. The first WB (left) shows eIF3f expression, detected with a polyclonal rabbit anti-human eIF3f antibody (primary antibody) and HRP-conjugated goat anti-rabbit antibody (secondary antibody). To document equal protein loading, the second WB (right) shows tubulin expression, detected with a monoclonal mouse anti-human beta-tubulin antibody (primary antibody) and HRP-conjugated goat anti-mouse antibody (secondary antibody). Click here for file

Additional file 8 **Amplified micrographs for the data used in Figure **5B. Amplified micrographs (~2 ×) of A549 cells transfected with pMSG (control) and pFH11S (ectopic expression). Micrographs were reduced to 40% of original size. Microscope final magnification: 40×. Click here for file

Additional file 9 **Original Western blots for the data used in Figure **6A**and **6B. Original Western blots (WB) for the analysis of eIF3f expression in transfected A549 cultures with pMSG (control), pFH11A (antisense), scrambled siRNAs (control), and eIF3f siRNAs. A549 cells were lysed 48 hours after transfection and proteins were transferred to a PVDF membrane after separation by SDS-PAGE gel electrophoresis. The first WB (left) shows eIF3f expression, detected with a polyclonal rabbit anti-human eIF3f antibody (primary antibody) and HRP-conjugated goat anti-rabbit antibody (secondary antibody). To document equal protein loading, the second WB (right) shows tubulin expression, detected with a monoclonal mouse anti-human beta-tubulin antibody (primary antibody) and HRP-conjugated goat anti-mouse antibody (secondary antibody). Click here for file

Additional file 10 **Amplified micrographs for the data used in Figure **6C. Amplified micrographs (~2 ×) of A549 cells transfected with scrambled siRNAs (control) and eIF3f siRNAs. Micrographs were reduced to 40% of original size. Microscope final magnification: 40×. Click here for file
