# Supplementary figures and images for: Expression of human eukaryotic initiation factor 3f oscillates with cell cycle in A549 cells and is essential for cell viability
Source: Cell Div. 2010 May 13;5:10. doi: 10.1186/1747-1028-5-10 (PMC2877012; doi:10.1186/1747-1028-5-10)

eIF3f  
(~ 47 kDa) →

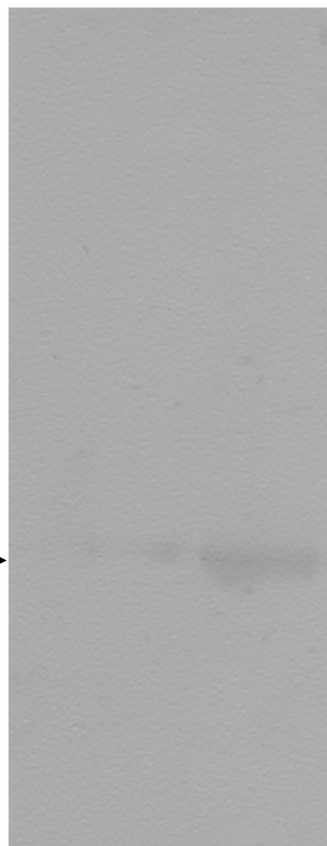

**G0**   **A**

← Tubulin  
(~ 55 kDa)

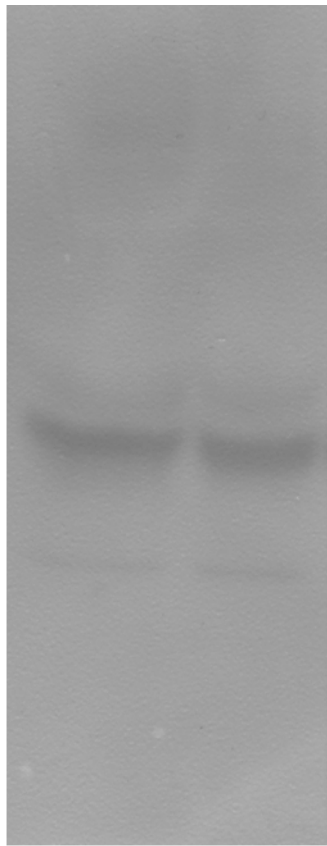

**G0**   **A**

Supplement: Additional file 1 — Original Western blots for the data used in Figure 1A. Original Western blots (WB) for the analysis of eIF3f expression in quiescent A549 cells (G0) and proliferating asynchronous cultures (A). The first WB (left) shows eIF3f expression, detected with a polyclonal rabbit anti-human eIF3f antibody (primary antibody) and HRP-conjugated goat anti-rabbit antibody (secondary antibody). To document equal protein loading, the second WB (right) shows tubulin expression, detected with a monoclonal mouse anti-human beta-tubulin antibody (primary antibody) and HRP-conjugated goat anti-mouse antibody (secondary antibody). [file 1747-1028-5-10-S1.PDF]

## eIF3f

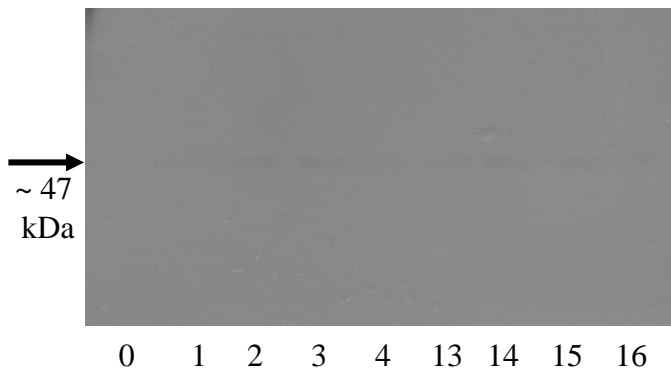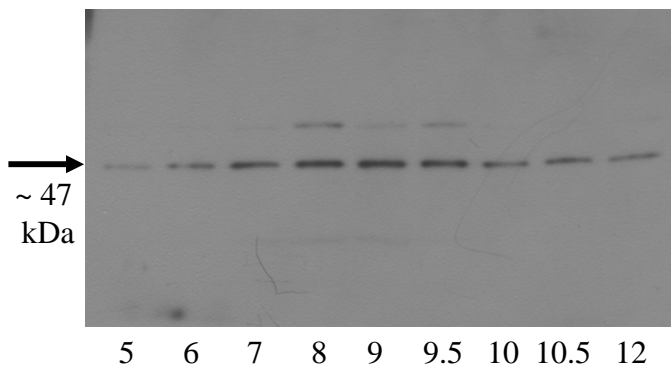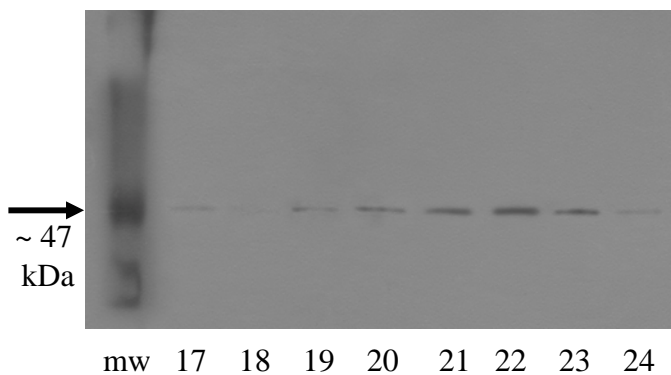

## Tubulin

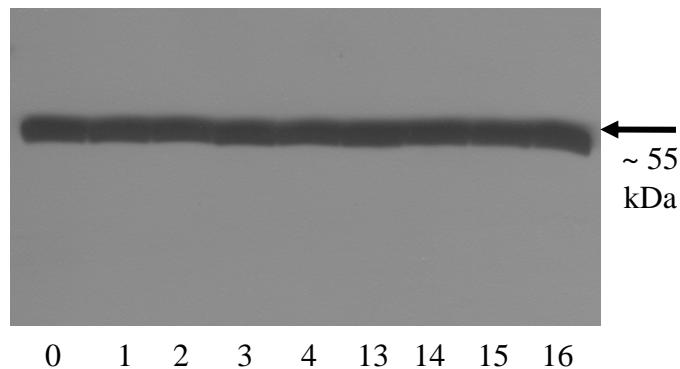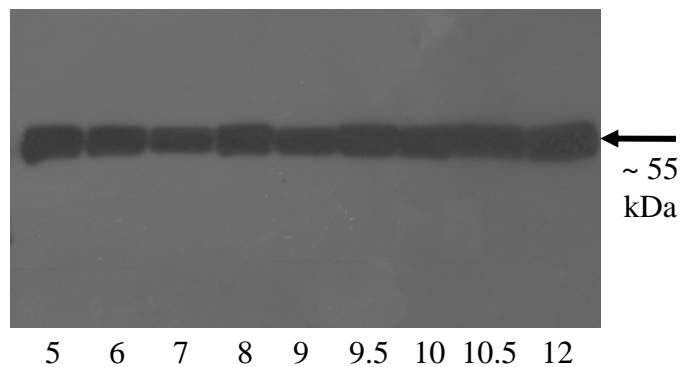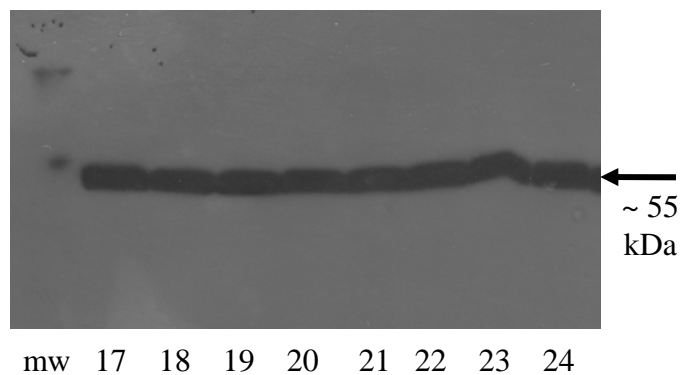

Supplement: Additional file 2 — Original Western blots for the data used in Figure 3A. Original Western blots (WB) for the analysis of eIF3f and tubulin expression in A549 synchronous cultures. The left WBs show eIF3f expression, detected with a polyclonal rabbit anti-human eIF3f antibody (primary antibody) and HRP-conjugated goat anti-rabbit antibody (secondary antibody). To document equal protein loading, the WB at the right show tubulin expression, detected with a monoclonal mouse anti-human beta-tubulin antibody (primary antibody) and HRP-conjugated goat anti-mouse antibody (secondary antibody). Numbers represent the time in hours after HU release in A549 cultures. (mw: protein molecular weight). [file 1747-1028-5-10-S2.PDF]

eIF3f  
(~ 47 kDa) →

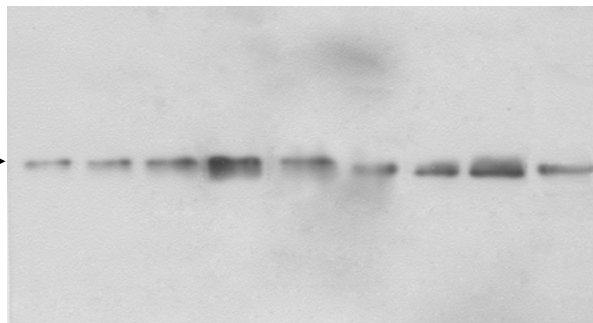

0 3 5 9 12 15 19 22 24

← Tubulin  
(~ 55 kDa)

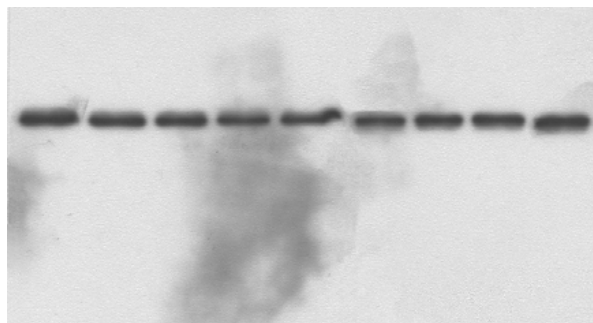

0 3 5 9 12 15 19 22 24

Supplement: Additional file 3 — Original Western blots for the eIF3f and tubulin data used in Figure 3B. Original Western blots (WB) for the analysis of eIF3f and tubulin expression in A549 synchronous cultures. The top WB shows eIF3f expression, detected with a polyclonal rabbit anti-human eIF3f antibody (primary antibody) and HRP-conjugated goat anti-rabbit antibody (secondary antibody). To document equal protein loading, the WB at the bottom shows tubulin expression, detected with a monoclonal mouse anti-human beta-tubulin antibody (primary antibody) and HRP-conjugated goat anti-mouse antibody (secondary antibody). Numbers represent the time in hours after HU release in A549 cultures. [file 1747-1028-5-10-S3.PDF]

## Cyclin B1

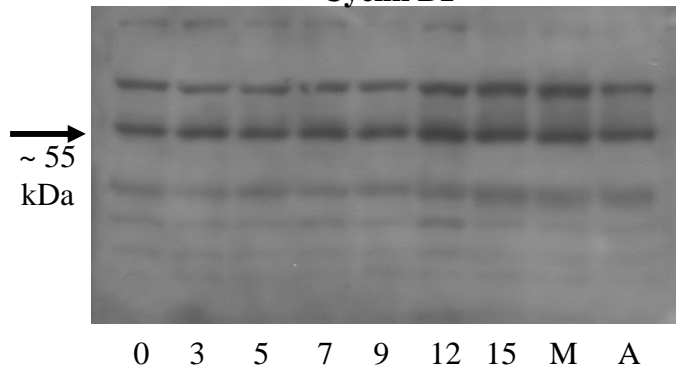

## Tubulin

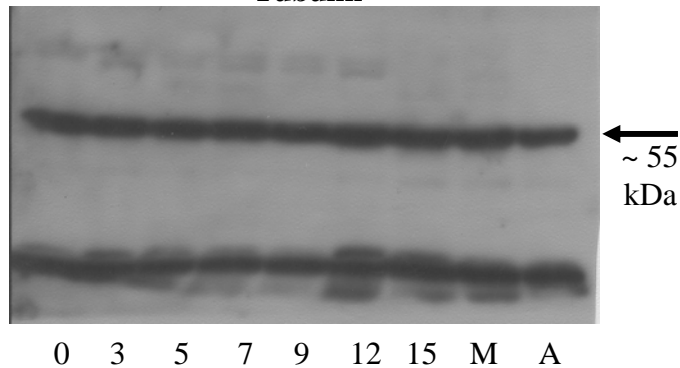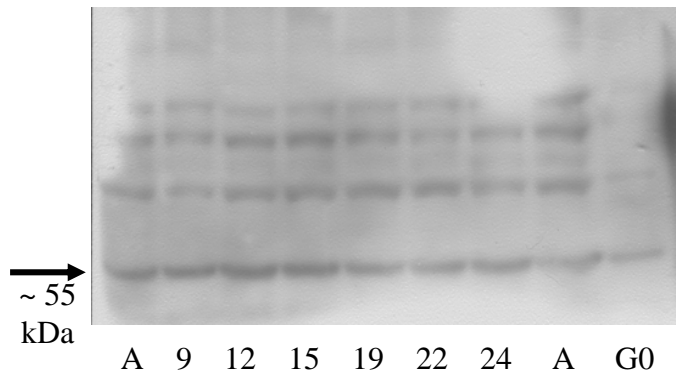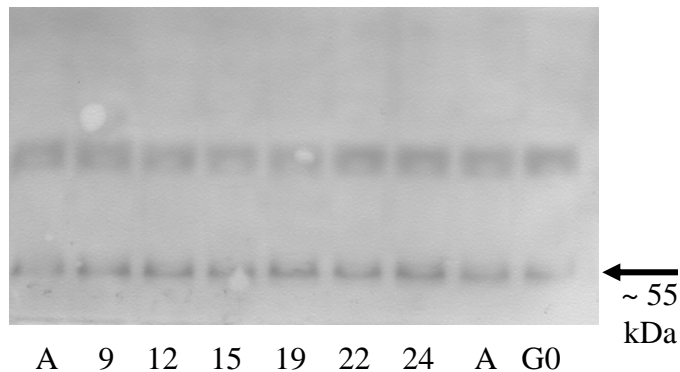

Supplement: Additional file 4 — Original Western blots for the Cyclin B1 data used in Figure 3B. Original Western blots (WB) for the analysis of Cyclin B1 expression in A549 synchronous cultures. The left WBs show Cyclin B1 expression, detected with a monoclonal mouse anti-Cyclin B1 antibody (primary antibody) and HRP-conjugated goat anti-mouse antibody (secondary antibody). To document equal protein loading, the WBs at the right show tubulin expression, detected with a monoclonal mouse anti-human beta-tubulin antibody (primary antibody) and HRP-conjugated goat anti-mouse antibody (secondary antibody). Numbers represent the time in hours after HU release; M represents expression in mitotic A549 cells, by accumulating cells with nocodazole; A represents expression in A549 proliferating asynchronous cultures; G0 represents expression in quiescent A549 cells, by accumulating cells with serum deprivation; and mw, protein molecular weight. [file 1747-1028-5-10-S4.PDF]

**A**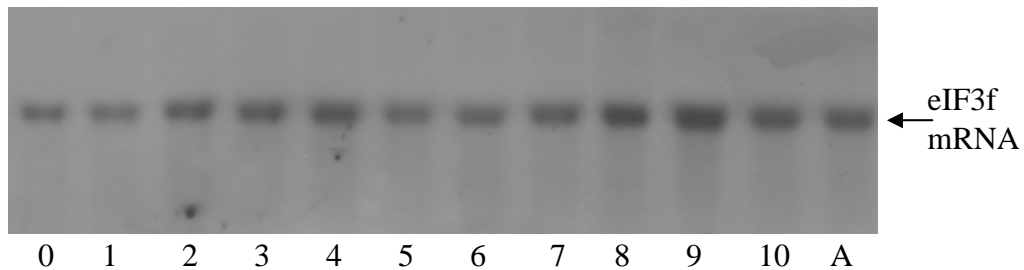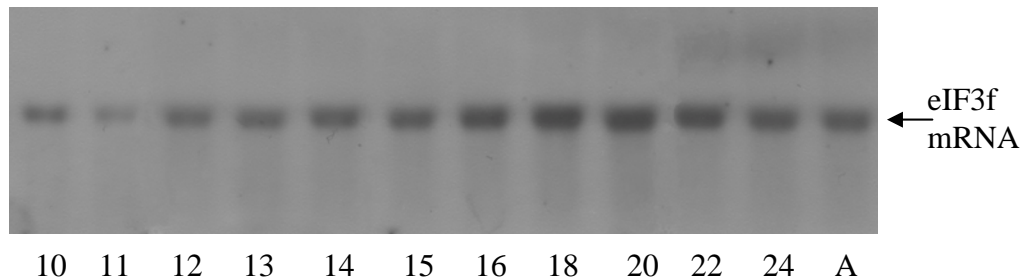**B**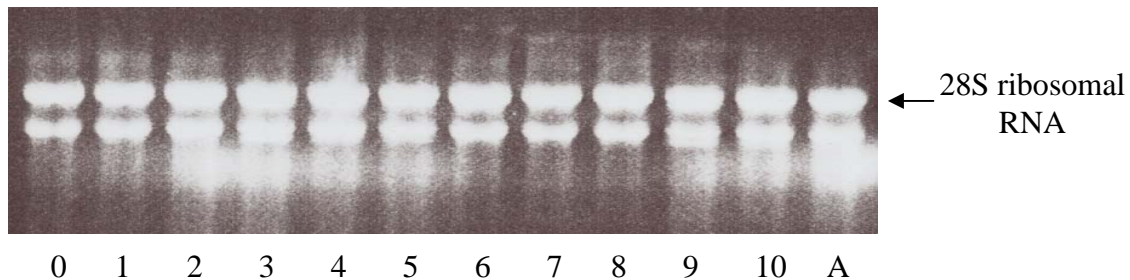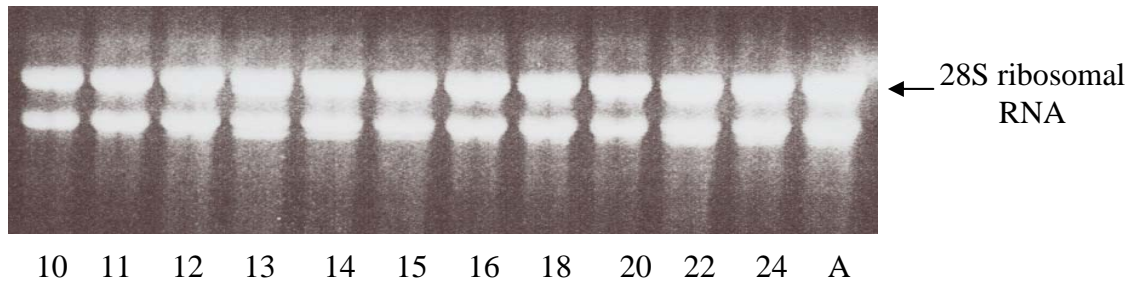

Supplement: Additional file 5 — Original Northern blots for the data used in Figure 3C. A) Original Northern blots (NB) for the analysis of eIF3f mRNA expression in A549 synchronous cultures. The NBs show eIF3f mRNA expression, detected with a complementary antisense single-stranded RNA probe of human eIF3f. To document equal RNA loading and to normalize the expression of eIF3f mRNA, the relative intensity units of the 28S ribosomal RNA bands were determined. B) Total RNA gel photographs, corresponding to the NBs shown in panel A, and taken with a Hoefer Photoman Polaroid Camera (Hoefer Inc., Holliston, MA). Numbers represent the time in hours after HU release, and A represents expression in A549 proliferating asynchronous cultures. [file 1747-1028-5-10-S5.PDF]

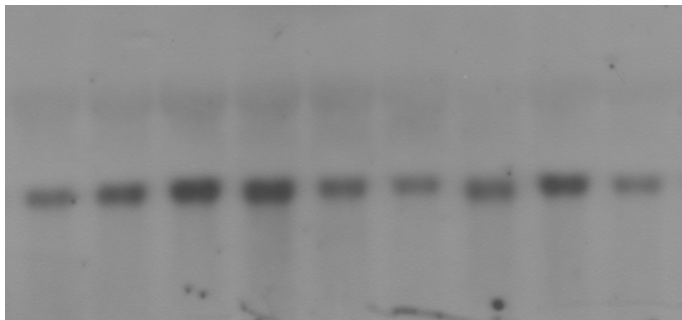

3 5 8 9 12 15 18 20 24

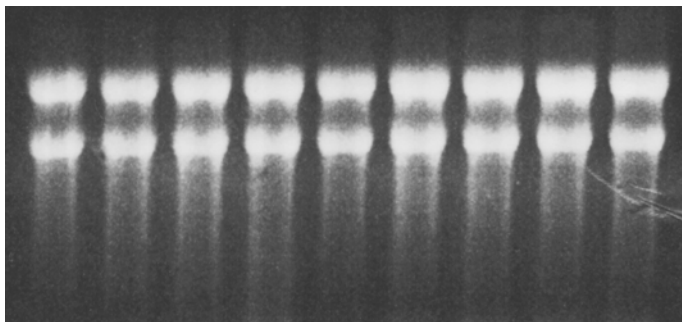

3 5 8 9 12 15 18 20 24

Supplement: Additional file 6 — Original Northern blots for the data used in Figure 3D. Original Northern blot (NB) for the analysis of eIF3f mRNA expression in A549 synchronous cultures. The NB (top) shows eIF3f mRNA expression, detected with a complementary antisense single-stranded RNA probe of human eIF3f. To document equal RNA loading and to normalize the expression of eIF3f mRNA, the relative intensity units of the 28S ribosomal RNA bands were determined. Total RNA gel photograph (bottom) corresponds to the NB shown above, and taken with a Hoefer Photoman Polaroid Camera (Hoefer Inc., Holliston, MA). Numbers represent the time in hours after HU release. [file 1747-1028-5-10-S6.PDF]

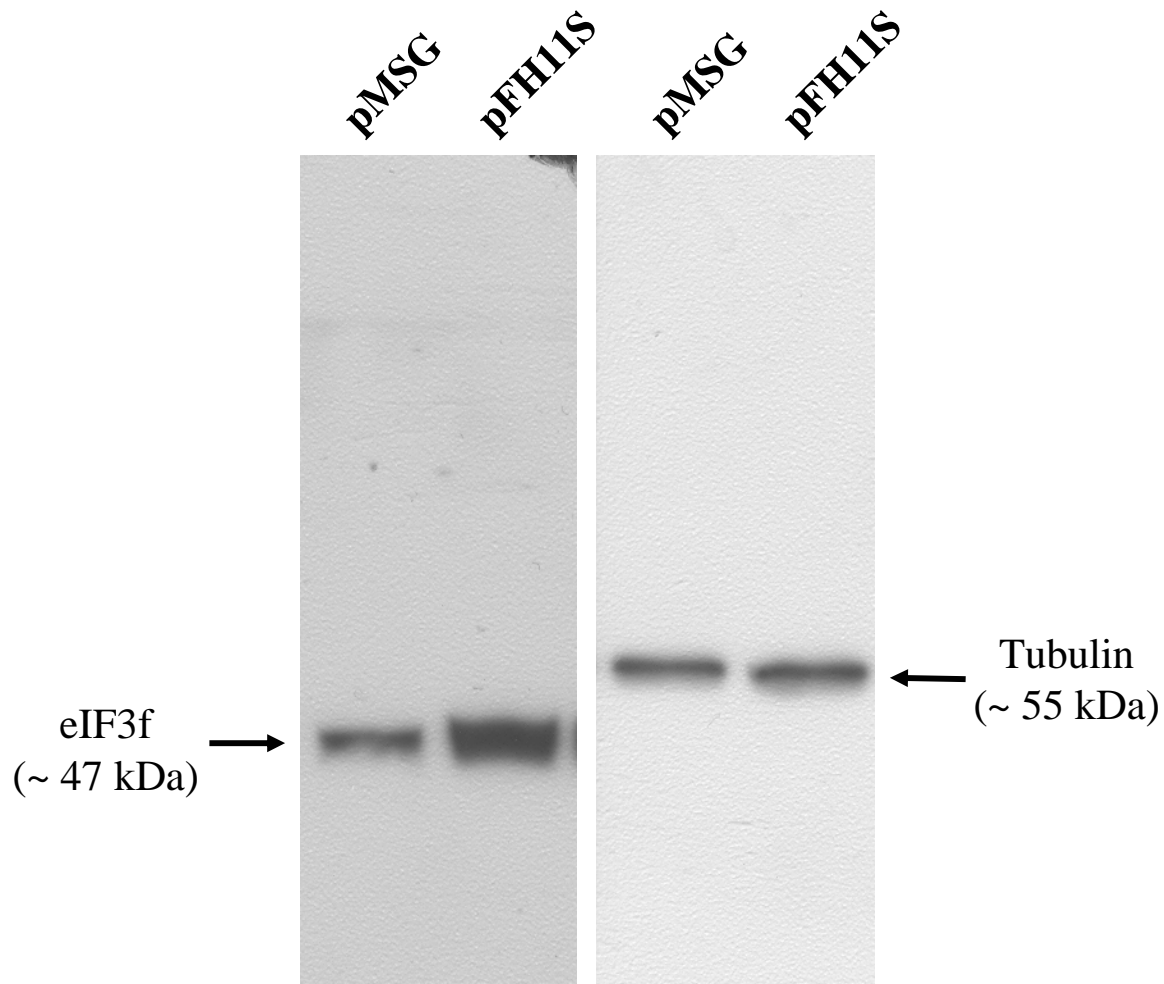

Supplement: Additional file 7 — Original Western blots for the data used in Figure 5A. Original Western blots (WB) for the analysis of eIF3f expression in A549 cultures transfected with pMSG (control) and pFH11S (ectopic expression). A549 cells were lysed 48 hours after transfection and proteins were transferred to a PVDF membrane after separation by SDS-PAGE gel electrophoresis. The first WB (left) shows eIF3f expression, detected with a polyclonal rabbit anti-human eIF3f antibody (primary antibody) and HRP-conjugated goat anti-rabbit antibody (secondary antibody). To document equal protein loading, the second WB (right) shows tubulin expression, detected with a monoclonal mouse anti-human beta-tubulin antibody (primary antibody) and HRP-conjugated goat anti-mouse antibody (secondary antibody). [file 1747-1028-5-10-S7.PDF]

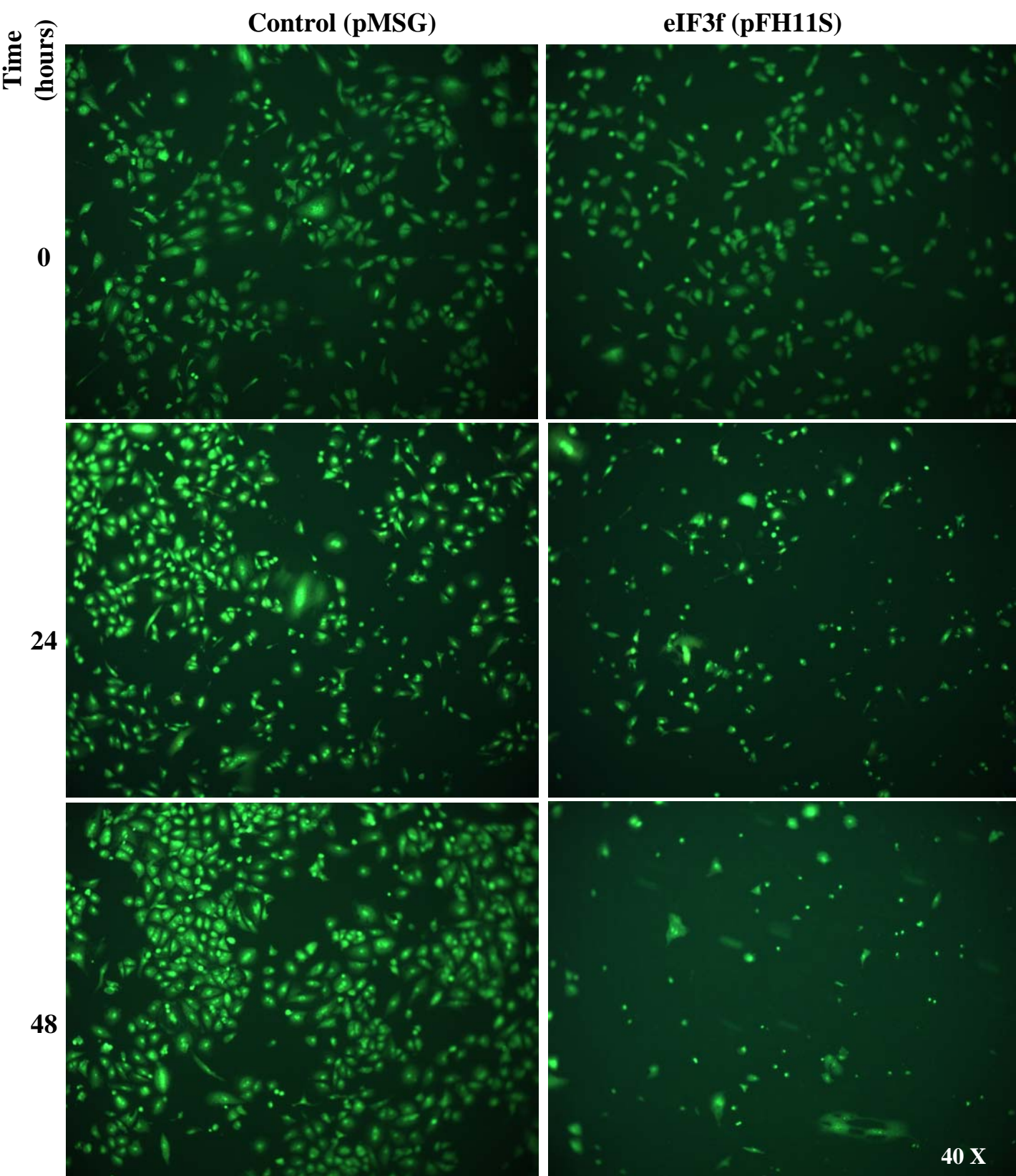

Supplement: Additional file 8 — Amplified micrographs for the data used in Figure 5B. Amplified micrographs (~2 ×) of A549 cells transfected with pMSG (control) and pFH11S (ectopic expression). Micrographs were reduced to 40% of original size. Microscope final magnification: 40×. [file 1747-1028-5-10-S8.PDF]

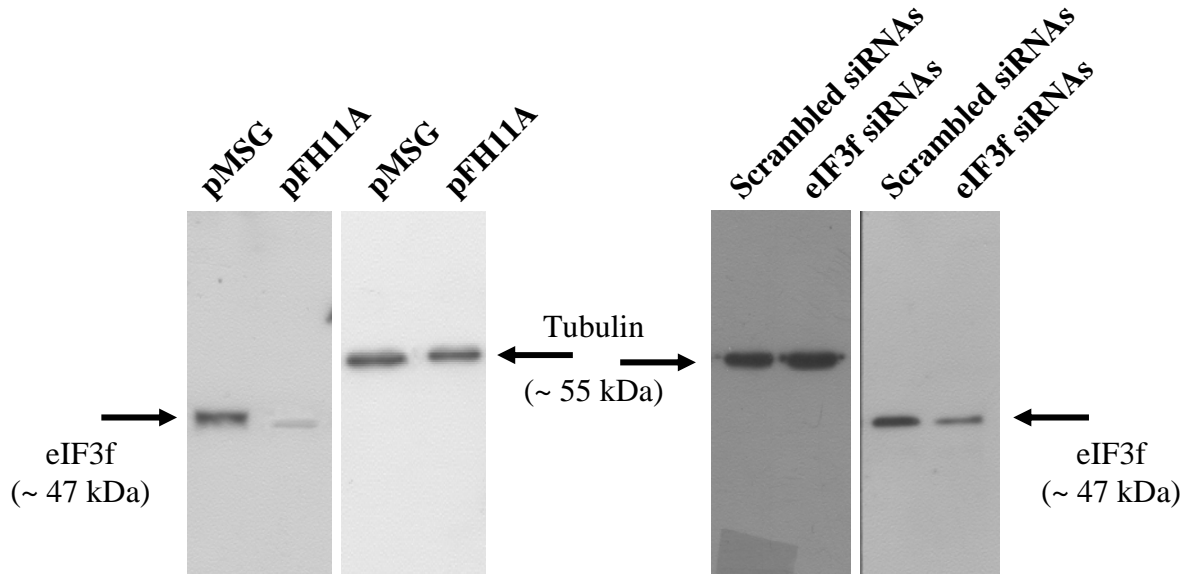

Supplement: Additional file 9 — Original Western blots for the data used in Figure 6Aand 6B. Original Western blots (WB) for the analysis of eIF3f expression in transfected A549 cultures with pMSG (control), pFH11A (antisense), scrambled siRNAs (control), and eIF3f siRNAs. A549 cells were lysed 48 hours after transfection and proteins were transferred to a PVDF membrane after separation by SDS-PAGE gel electrophoresis. The first WB (left) shows eIF3f expression, detected with a polyclonal rabbit anti-human eIF3f antibody (primary antibody) and HRP-conjugated goat anti-rabbit antibody (secondary antibody). To document equal protein loading, the second WB (right) shows tubulin expression, detected with a monoclonal mouse anti-human beta-tubulin antibody (primary antibody) and HRP-conjugated goat anti-mouse antibody (secondary antibody). [file 1747-1028-5-10-S9.PDF]

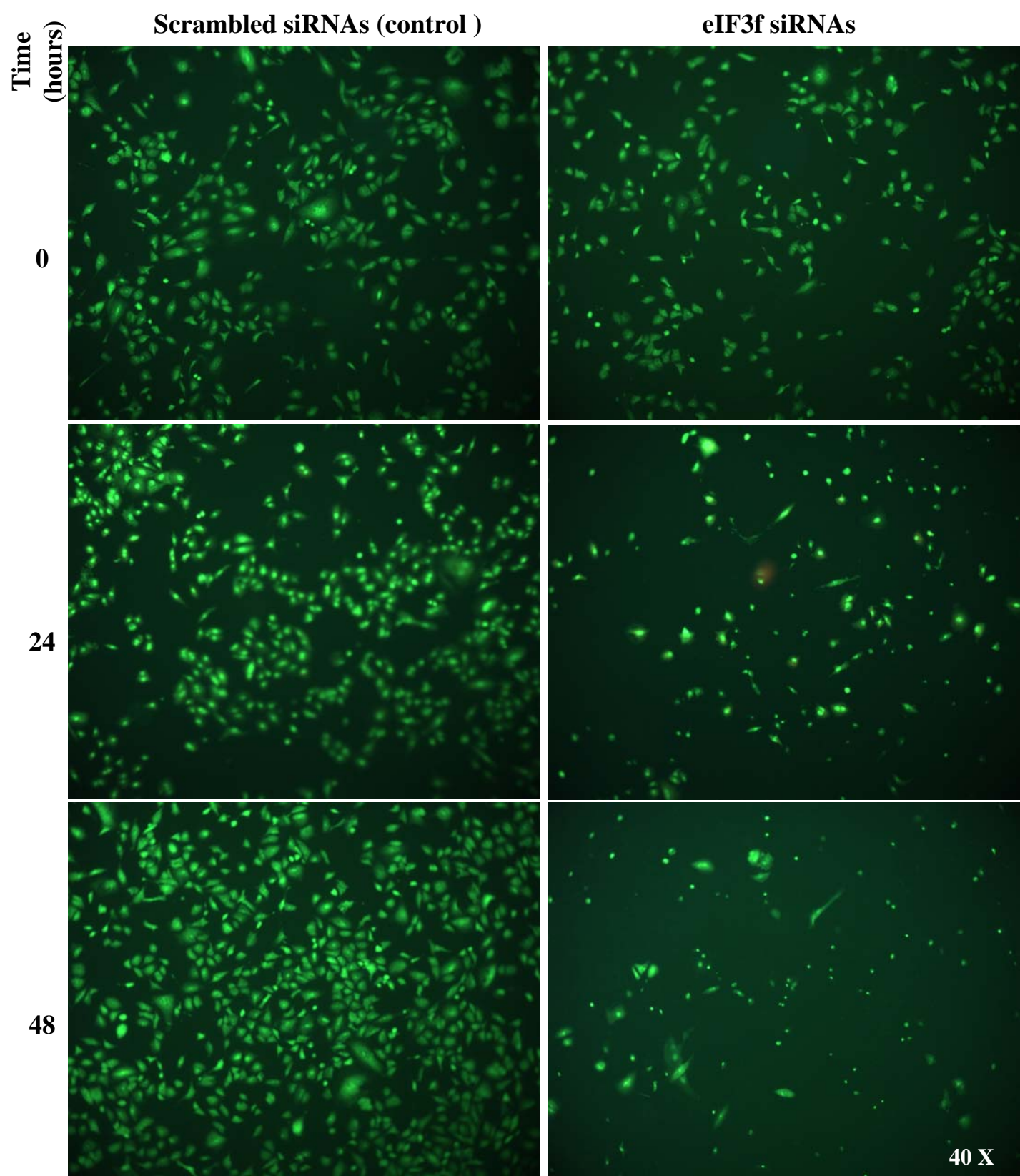

Supplement: Additional file 10 — Amplified micrographs for the data used in Figure 6C. Amplified micrographs (~2 ×) of A549 cells transfected with scrambled siRNAs (control) and eIF3f siRNAs. Micrographs were reduced to 40% of original size. Microscope final magnification: 40×. [file 1747-1028-5-10-S10.PDF]
